# A New Immersive Virtual Reality Station for Cardiopulmonary Resuscitation Objective Structured Clinical Exam Evaluation

**DOI:** 10.3390/s22134913

**Published:** 2022-06-29

**Authors:** Manuel Rodríguez-Matesanz, Carmen Guzmán-García, Ignacio Oropesa, Javier Rubio-Bolivar, Manuel Quintana-Díaz, Patricia Sánchez-González

**Affiliations:** 1 Biomedical Engineering and Telemedicine Centre, ETSI Telecomunicación, Centre for Biomedical Technology, Universidad Politécnica de Madrid, 28040 Madrid, Spain; manuel.rodriguez.matesanz@upm.es (M.R.-M.); carmen.guzmang@upm.es (C.G.-G.); i.oropesa@upm.es (I.O.); 2 Hospital Universitario La Paz de Madrid, 28046 Madrid, Spain; javier84.rubio@gmail.com (J.R.-B.); mquintanadiaz@gmail.com (M.Q.-D.); 3 Centro de Investigación Biomédica en Red en Bioingeniería, Biomateriales y Nanomedicina, Instituto de Salud Carlos III, 28029 Madrid, Spain

**Keywords:** virtual reality, objective structured clinical exam, cardiopulmonary resuscitation, surgical simulation, chain of survival, OSCE

## Abstract

The Objective Structured Clinical Exam (OSCE) is an assessment tool used as a reliable method for clinical competence evaluation of students. This paper presents an investigation focused on the chain of survival, its related exploration, management, and technical skills, and how Virtual Reality (VR) can be used for the creation of immersive environments capable of evaluating students’ performance while applying the correct protocols. In particular, the Cardiopulmonary Resuscitation (CPR) procedure is studied as an essential step in the development of the chain of survival. The paper also aims to highlight the limitations of traditional methods using mechanical mannequins and the benefits of the new approaches that involve the students in virtual, immersive, and dynamic environments. Furthermore, an immersive VR station is presented as a new technique for assessing CPR performance through objective data collection and posterior evaluation. A usability test was carried out with 33 clinicians and OSCE evaluators to test the viability of the presented scenario, reproducing conditions of a real examination. Results suggest that the environment is intuitive, quick, and easy to learn and could be used in clinical practice to improve CPR performance and OSCE evaluation.

## 1. Introduction

The Objective Structured Clinical Exam (OSCE) is a versatile method to evaluate the competence of students and clinic professionals. The examined trainees interact with a series of simulated patients in scenarios called stations, where their competencies are evaluated through direct observation by a team of examiners [1]. Students are observed and qualified while performing in the different stations, obtaining a score that is not affected by prejudice [2].

The only training method that existed until the end of the 90s and that continues to be executed as the standard way of OSCE evaluation is the one carried out through face-to-face courses imparted by an expert OSCE instructor, who also acts as an examiner, using physical mannequins [3]. These courses have some drawbacks. On the one hand, the implementation may not be realistic enough, and on the other hand, the evaluation can be subjective since some parameters are difficult to quantify [3]. The latter has been addressed in recent years by using electronic devices capable of gathering appropriate parameters and metrics [4]. Thus, examiners can use the generated metrics (see Table 1) to give an objective assessment [4]. Depending on where they are placed, these devices are divided into two categories: (1) those worn by the student, which are usually placed on the student’s wrist and which provide visual [5] and auditory feedback on the screen and speakers of the device [6]; and (2) those integrated into the mannequin [7], which are more precise in terms of certain parameters (e.g., the world-space position and rotation of the body, the number and accuracy of repetitions, etc. [8]) but with a higher cost and a massive battery consumption [9].

One of the most popular OSCE stations is focused on the training and assessment of the chain of survival (CoS). The CoS is a sequence of actions performed to resuscitate the individual who is suffering and needs the help of a third party to breathe again or to get his heart beating again while suffering from an emergency.

It is estimated that between 40–50% of mortality is due to a cardiac arrest. Early defibrillation, as well as a correct and accurate execution of the Cardiopulmonary Resuscitation (CPR) procedure, are determining factors in the survival of those victims. CPR is a part of the CoS defined as the set of temporary and standardized compression maneuvers designed to ensure oxygenation of vital organs when a person’s blood circulation stops of a sudden, regardless of the cause of cardiorespiratory arrest. Therefore, it is considered essential that all present and future medical professionals apply it. This aspect, in line with the continuous development of the new technologies such as XR, in the field of medical education, indicates the need to develop tools that test the knowledge and skills of students in the execution of the BLS.

The first step for training in the CPR execution is the preparation of didactic material and evaluation elements in accordance with the guidelines of CPR scientific societies such as the European Resuscitation Council (ECR) [10] or the American Heart Association (AHA) [11]. These guidelines are updated every five years, so any didactic material relative to CPR must be reviewed once the updates are published [10].

The student learning process must follow an initial theoretical phase where knowledge about CPR is taught for later execution. For instance, the 30:2 maneuver involves interspersing two rescue breaths between periods of thirty thoracic compressions. The ERC recommends that the pause time for taking rescue breaths should not exceed ten seconds to ensure the security of the victim [10].

Assessment tests are given to confirm that the students have assimilated the knowledge correctly. Once this phase reaches the end, practical activities can be carried out to allow students to improve their technical skills related to CPR. The more students practice these protocols, the better outcome they achieve, increasing the survival probability of the victim [12]. Furthermore, the most effective training routines involve both learning and deliberate practice, as the trainee puts into action the assimilated knowledge, strengthening the retention of the skill and retrieving feedback from the student [13].

CPR has traditionally been trained and evaluated through the usage of mechanical mannequins [14]. This procedure, despite giving haptic feedback to the user, is not able to provide an immersive and dynamic environment on its own. In order to solve this, newer approaches involve situating the trainee into a scenario that simulates a real situation to make them move faster in the learning curve [14].

One of the key parts that differentiate the simulation from a traditional OSCE station is the environment (i.e., the development of a story to encompass the entire experience in which the student gets immersed) [15]. Traditionally, the first thing is to give the student a briefing on the starting situation. This briefing provides the student with a description of the scenario where the task takes place (e.g., where the emergency occurs, the victim’s age, etc.). This needs to be kept in mind when developing a VR application, as this description must be implicit, requiring the student to pay attention to the environment and providing them in-engine auditive instructions.

The potential of VR simulations, along with the technological advancement of devices in medical settings, are currently under research. In fact, they have turned into a new way of training and evaluation with fewer resource demands while maintaining the motivation of the examined students. This is because VR technology permits minimizing the intervention of the examiner while encouraging the user’s motivation, obtaining a smoother performance curve [16]. For instance, most of the simulators generate from five to ten of the metrics explained in Table 1 depending on the trained skill and the usage of the mannequin, and the simulation performance [10,17]. Additionally, the utilization of this technology allows the interaction with a full set of computer-generated objects in different virtual 3D scenarios that simulate real situations without any additional cost. Flexible engines such as Unity Engine supply a quick and easy-to-use experience in terms of 3D application development [18]. Furthermore, thanks to the variety of programmable scenarios within a VR environment, the learning curve is higher due to the variety and correct application of the protocol in each case.

In the last two decades, technological systems have evolved into more advanced systems capable of creating virtual environments in which realistic, efficient, and safe scenarios are simulated [19]. This approach has already been applied in multiple fields of medicine, such as internal medicine or surgery [19]. These scenarios can be used to analyze additional aspects on top of the metrics provided by instrumental feedback devices.

The use of virtual environments began with the development of hybrid applications called Mini-Virtual Reality Enhanced Mannequins (Mini-VREM). Mini-VREMs offer non-immersive simulations, combining the use of auditory and visual stimuli with real-time feedback provided by the physical mannequin [20]. Some simulators in this category are RELIVE [21], a mobile application connected to a mannequin that tracks the execution of the patient or LISSA, a desktop application that makes use of the Microsoft Kinect for a full body tracking system [22].

Virtual Reality has been used numerous times for the practice of CPR. The newest methods offer a high-fidelity simulation and experimental learning based on extended reality technologies. Simulators include CODE-BLUE [23], which provides a fully immersive simulation of cardiac arrest on the public road; VR-CPR [24], which simulates the mechanical behavior of the patient’s chest and the AED during CPR performance; VR-UCAM, which provides a computer-generated environment where external feedback devices are applied in the simulation [25]; or LifeSaver, a United Kingdom council simulator which evaluates the performance of CPR [26].

On the other hand, there are examples such as VR-Hybrid, which tries not only to focus on the learning and training of the students but also on the assessment. This simulator has three development modes:

1. Learning. The student follows a guided procedure to practice a specific skill.
2. Training. The student receives real-time feedback and audio-visual stimulus to test the concentration.
3. Assessment. The student faces a CPR test without any guide and receives real-time feedback from the physical mannequin.

More recent studies highlight approaches focused on gamification features such as leader boards and difficult progressive systems to incentive the trainees to encourage repeated training while receiving real-time feedback in first-person perspective simulations of a cardiac arrest patient [27]. Similar to CPR-OSCE VR, one more VR system was developed in 2019, focusing on showing the potential benefits of immersive VR through feedback and gamification and using a standard CPR mannequin, hand-tracking, and a VR headset [28]. In addition, another study explores the usage of a VR mobile training app to see if bystander response could be improved in a CPR emergency situation, making use of Google Cardboard as the VR headset [29].

Table 2 shows a comparison of the abovementioned simulators based on the evaluation of the CPR and the feedback they provide to the user while it is performing following the AHA guidelines.

As observed in the analysis of the simulators currently available in the market, the availability of evaluation mode is rare, as well as the provision of both summative and formative feedback. Hence, one of the main aims of this study is to develop a new VR-based solution where CPR performance is trained and assessed by means of the collection of objective data: CPR-OSCE VR.

Moreover, the technological challenge of implementing a cross-platform simulation capable of being played on cheap wireless headsets and computer devices with a more precise evaluation has been taken into account for the development of the abovementioned VR-based solution. Thus, CPR-OSCE VR does not only aim to evaluate the CPR technique with a reproducible and objective method but also offers a realistic scenario with audio-visual feedback where each student can practice and improve their CPR technique to obtain better results in each iteration while approaching the accessibility of the system to easy-to-access devices.

## 2. Materials and Methods

This article presents a simulator defined as CPR-OSCE VR, which allows evaluating OSCE students with objective data collection. The trainee interacts with a patient that suffers a cardiac arrest in real-time in a real-world scenario such as a train station. The world-space position and rotation of the patient in the VR workspace are determined by a tracked device attached to a physical mannequin. In this system, the accuracy of the performance of the algorithm is based on the physics collisions between the user’s hands, tracked by the Head-Mounted Display (HMD) controllers, and the collision bounding primitives of the different objects in the environment. These collisions grant the system the ability to calculate the frequency, accuracy, and depth of the chest compressions. In addition to physics-based interaction, speech recognition plays an important role in the simulation. Calling emergency services is required to complete the Basic Life Support (BLS), a modality of the CPR training procedure which consists of a sequence of steps to follow for the treatment of an unconscious adult victim using only the hands [3]. Within this framework, the simulation also uses a speech recognition module that waits for specific voice commands received from the microphone, if the computer has one available, to start recording audio from the microphone during the call. The recorded audio can be analyzed afterward in order to evaluate how adequate the call to emergency services is. Within the audio, some metrics are collected locally and saved to the computer for further visualization and examination. These metrics are classified according to their corresponding link in the chain of survival as well as their correspondence with the areas of competence that the OSCE evaluates (Table 1).

The following paragraphs describe (1) the architecture of the simulator, (2) the different modules and systems that compose the interactions and how they are adapted to the CPR procedure, and (3) the protocol implemented for the usability tests.

### 2.1. CPR-OSCE VR: Design and Implementation

#### 2.1.1. General Architecture

CPR-OSCE VR is a software application designed with a cross-platform approach in mind and can be executed both on PC or with Oculus Quest, which uses the Android Operating System. The simulator has been developed as an easy-to-use, intuitive, and portable way to measure objective data regarding CPR performance. Therefore, the trainee needs to have knowledge about the technique and the procedure they are about to perform, as well as the usage of the AED.

**Figure 1:**
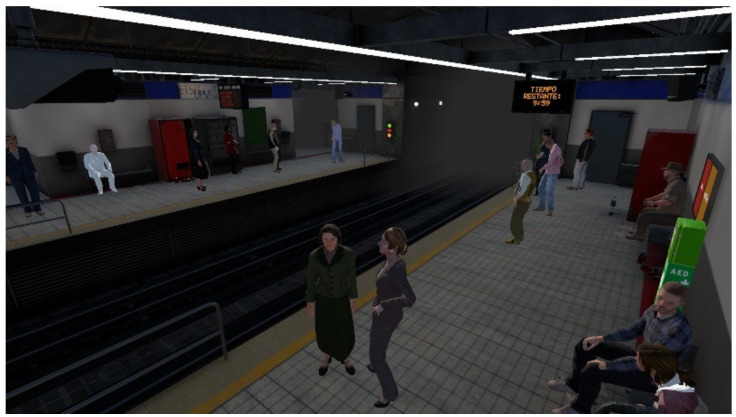
Scene composition based on the Metro of Madrid with assets created and animated in Blender and with static physics collisions baked into the scene.

The student embodies the role of a bystander in the BLS procedure performed on an elderly man who goes into cardiac arrest. By interacting with the immersive VR simulator without any additional material rather than their own hands if hand-tracking technology is activated in the simulator settings, given by a configuration CSV file, or the headset controllers. More specifically, to give experience with which to get easily comfortable, the chosen setting consists of a train station modeled akin to the Metro of Madrid, Spain. Moreover, these stations are usually crowded public spaces which have mandatory AED available for public use, as per the Community of Madrid legislation [23]. Hence, different props were developed in Blender (a free and open-source 3D modeling program) [30], as well as different prefabs based on static collisions and scene composition (Figure 1). This software was developed on the videogame engine Unity 3D (version 2021.1.9f1) and featured multiple scripts to visualize the different environment props and characters, track the user’s location, movement, and interaction, and measure the accuracy of the CPR performance.

The system can be used both with and without an external physical mannequin and the extra tracking device. The mannequin is positioned on the floor at the beginning of the simulation. The position in the world-space is tracked by the external tracker device, and then the position is mapped to the 3D model of the victim in the simulated local workspace. In the case it is not desired to use the mannequin, the right-hand controller can be used to position the victim in the workspace while using the left-hand controller for interacting with the environment. Furthermore, if the former is using an Oculus Quest 2 as HMD, the Oculus hand-tracking can be set up to the hands during the simulation using the right-hand controller as victim position setter. In any case, the real workspace must be empty and with enough space to walk around. When the 3D victim suffers a cardiac arrest, the model ends in a similar position to the real mannequin (which is already on the floor). The VR devices automatically show by default a virtual wall when the user is near the limits of the mentioned workspace.

Internally, the CPR-OSCE VR uses a configuration .csv file which is included in the same folder as the executable files and is accessed by asynchronous resource loading. This file stores several parameters that are serialized into the internal data used by the system components and that need to be configured by the instructor prior to the student’s performance (i.e., adding the name and ID of the student and the duration time). In the case of standalone devices such as Oculus Quest, the input file is stored as a template in the application’s internal resources and needs to be filled by the student with an in-game menu. The data manager reads the default inputs from the template file and serializes the data into the application manager. After the completion of the exercise, this original identification data are moved to a different externally saved file where the objective metric data about the performance is stored.

**Figure 2:**
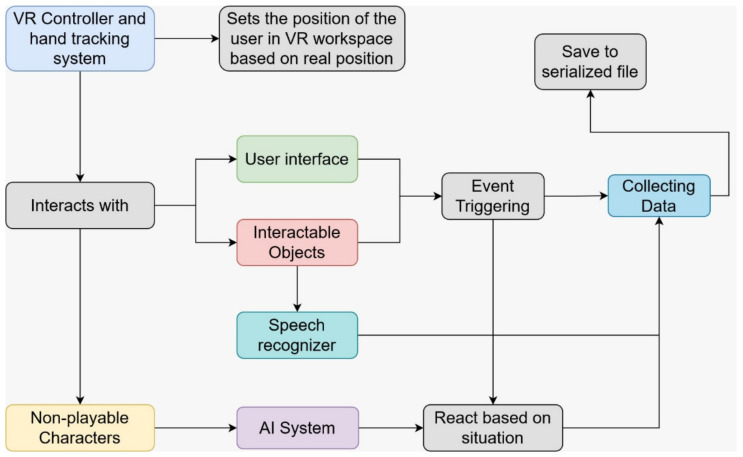
CPR OSCE VR workflow based on the simulator architecture and how components work during the simulation.

The simulator architecture, summarized in Figure 2, includes the following modules: environment, adaptation scene, application settings, custom event system, victim behavior, characters’ artificial intelligence (AI), speech recognition, User Interface (UI), extended reality (XR) tracking and interaction and application management. Unity applications are based on virtual object compositions called scenes. Each object relates with others thanks to attached behavior scripts called “components”, so the different modules are a composition of components that relate between them during the simulation.

#### 2.1.2. Services-Based Singleton Pattern

Singleton is of the most popular patterns in Unity development thanks to the usage of Microsoft’s .NET framework by the engine, the simplistic nature of the pattern, and the ease of implementing it together with design features such as *Don’t Destroy On Load* (DDOL), which avoids the memory deallocation of specific UnityEngine.Object instances when loading a new scene [31,32] DDOL is regularly used for scripts in which instances need to persist during the execution of the application, such as managers. However, as Unity does not allow the instances’ reference access from across the scenes, global instances are required. However, if not implemented properly, Singleton pattern leads to fault-prone code designs, and manual scripting order must be established in the project configuration to avoid thread races and undesired null pointer errors. Because of this, a service layer was implemented while keeping singleton instances with DDOL. Every management behavior (resource loading and unloading, audio playing, AI behaviors, input handling, or body tracking and positioning) is considered a service and centralized in a scriptable object that returns the instance based on the required service. For example, when audio is loaded into the audio source to be played, an audio manager is accessed by the audio service to handle the data such as the volume, pitch, frequency, or 3D audio threshold. If a service is not already initialized but required, this central manager creates a new instance, initializes the correct data, and returns it to the object to continue the application.

#### 2.1.3. Scene Management and Asset Bundles

**Figure 3:**
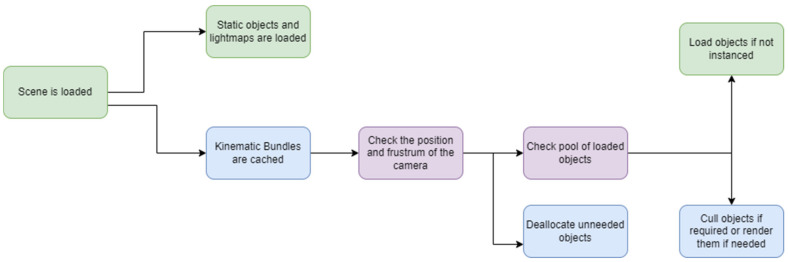
Bundle loading.

CPR-OSCE VR targets not only PCVR with HTC Vive but Oculus Quest 2 in standalone mode, which limits the number of rendered polygons, managed vertices, and calculated lightmap sizes to maintain the 72–90 Hz framerate in the regular mode, 120 Hz in the “Boost” mode. Therefore, a custom resources management system was implemented to load objects and free the allocated memory when required, similar to Unity’s Asset Bundles. Asset Bundles are archive files that contain platform-specific non-code Assets such as models, textures, prefabs, audio clips, or even entire scenes; that Unity can load at runtime while maintaining the dependencies to the rest of the system and capable of being delivered in an efficient compressed way [33]. CPR-OSCE VR bundles are compressed with the lossless compression algorithm called LZ4. This method provides speed superior to 500 MB/s compression and 1.5 GB/s decompression per core, scalable with multi-cores CPU [34]. These asset bundles are stored in the application’s Streaming Assets folder and referenced by GUI hash in configuration Scriptable Objects, which are data containers used to save large amounts of data by reducing the project’s memory usage by avoiding copies of values [35]. The scenes’ setup is based on what objects need to be loaded in runtime, depending on where the simulation is taking place. For example, the first scene bundle is automatically loaded, and the main managers and non-interactable objects such as walls, floors, and static props are loaded in memory while the splash screen is being faded. This scene is never unloaded as it contains the common scripts and objects to the whole simulation. Then, specific tags are managed by the simulation depending on the actions of the user to know what hashes are required. The resources manager allocates and load non-static objects into the scene considering the camera position and the camera frustum and what is being culled or near to be culled so distant objects are not considered to be rendered, based on distance threshold defined in the bundle prefab and the current occlusion culling if those are not located between the near and the far planes of the frustum or behind other polygons, applying the back-face culling; are unloaded [36] (Figure 3).

This method also applies to characters but saves the temporary state data (transform matrix values, animation key values, and object state). The animator’s current state is saved in the temporary data while being culled and resumes the animation from the paused keyframe once it is being rendered again. Every object is considered culled when one polygon of the mesh is being rendered, so objects with more than one sub-mesh that are rendered completely are baked into one joined mesh avoiding ass many batches in the same render pass as possible. In addition, loaded bundle references are cached in a stored pool for faster access. Because of this approach, multiple scenarios can be created while sharing the same system as new model, audio, texture, and prefab bundles are required.

#### 2.1.4. Rendering Optimizations

Non-stable framerate is one of the main factors in the motion sickness appearance while performing simulations in VR [37]. The increased realism in graphics and physics interactions is useless if the performance is affected by massive frame drops. In recent years, rendering optimization algorithms such as AMD’s Fidelity Super Resolution (FSR) [38] and Application Space-Warp (AWS) [39] were developed to predict new frames and interpolate the result, maintaining the framerate when heavy loading is being performed in XR devices. CPR-OSCE VR implements AWS by default if Oculus Quest 2 is being used but permits the user to select the optimization technique in the simulation configuration settings. In this case, the simulator renders a motion vector buffer and a depth buffer, in addition to the standard eye buffer, which the Oculus system will then use to synthesize new frames and still output maximum framerate per second to the display (72 in regular mode).

On the other hand, FSR uses temporal algorithms to reconstruct fine geometric and texture detail with anti-aliased output for smoother edges while keeping a lower resolution. When this is applied to CPR-OSCE VR, the output is lowered in resolution and then upscaled to the 1920 × 1832 resolution per eye. Using any of both algorithms allows the simulator to render approximately four hundred thousand polygons in real time while maintaining the frame rate.

#### 2.1.5. Control

CPR-OSCE VR is prepared for a 2 × 1.5 m workspace with free physical movement of the user. However, as this might not be the case of use, user-friendly controls are added to the VR user controller. By using the joystick or D-PAD trackpad in the case of HTC Vive wands, smooth locomotion is applied to the user’s virtual avatar as if the real body was moving.

In order to control the user’s body and track the head and hands for multiple systems without changing the core of the simulator, the Virtual Reality Interaction Framework (VRIF) was used [40]. This framework has an automated wrapper that exploits Unity’s extended reality (XR) module for each platform, so not a single line of code must be rewritten to export to a different platform. The VRIF player controller prefab features grabbing 3D objects in the virtual space, interacting with world-space canvas (UI), and making use of a locomotion manager with smooth locomotion and teleport settings. The grabbing action is based on physic collisions considering the Unity’s physics layer matrix and rigid body component for applying forces to them. For example, to grab the AED (grabbable object), the user needs to get close to it and grab it with the Trigger button in the controller, while they should press the button up to drop it where desired.

**Figure 4:**
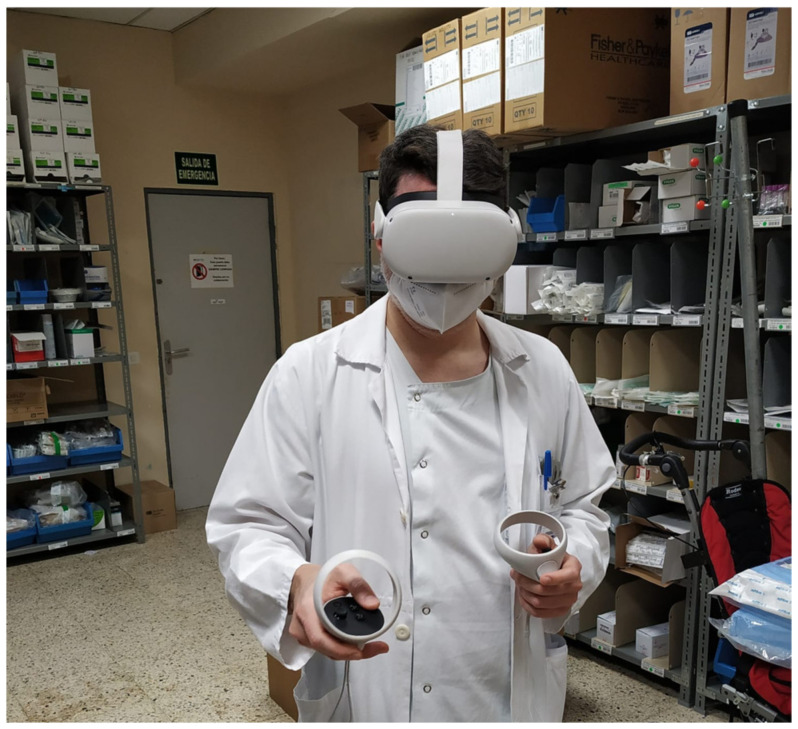
User interacting with the AED.

As Unity’s XR module supports oculus hand-tracking integration, in the case the user tests CPR-OSCE VR in Oculus Quest system, the Oculus hand tracking is available. In this case, the user needs to place the hands in front of the headset with the palms looking forward. The system automatically recognizes the hands and changes to hand-tracking mode so that users can interact with virtual objects with their own hands without requiring external resources (Figure 4). If the user presses any input in the regular Oculus Quest controllers, the controller mode is activated, and hand-tracking is disabled.

The smooth locomotion is disabled in hand-tracking mode, but the user can teleport by making an L-pose with the index and the thumb fingers with the palm facing up and moving the index finger towards the palm of the hand. A visual gizmo appears in real-time in the world-space showing where the user can teleport to. If a collision is detected in the teleport path, a red color line appears instead as a visual guideline.

**Figure 5:**
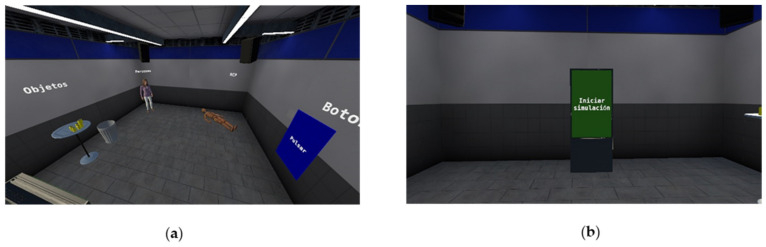
(**a**) Adaptation scene where the user can test the simulator mechanics. (**b**) User interface which triggers the start of the simulation once the user is ready.

All things considered, most users are not used to VR devices. For this reason, an adaptation scene was developed (Figure 5a) to allow users to experiment with the controls as well as the hand and head tracking. Users can interact with the different characters and objects found in the main simulation, such as grabbable objects, a physics-based body in which CPR can be practiced, non-playable characters that react to the user’s gestures, and some User Interface examples. Taking advantage of this first scene, the narrative of the situation and the instructions are presented to the student by auditory feedback. Users can listen to this information once but are able to start the simulation at any given moment by pressing a big start button in the middle of the scene (Figure 5b).

#### 2.1.6. Speech Recognition

**Figure 6:**
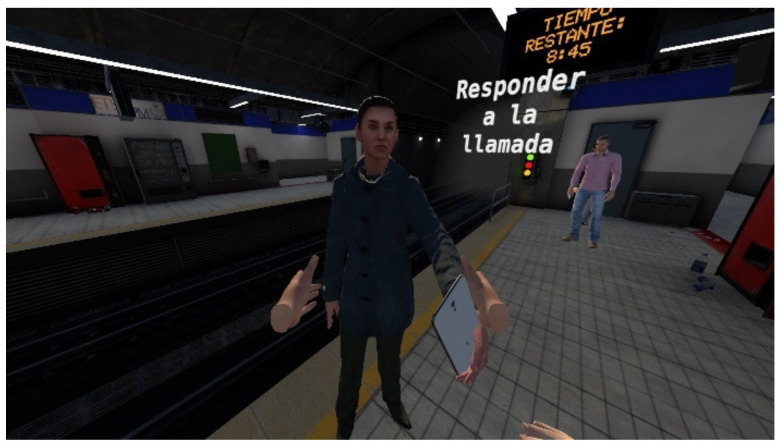
Character offering a mobile phone device, which needs be picked up by the user in order to activate the speech recognition to call the emergency services.

In addition to this, tasks are considered complete by the implementation of a speech recognition tool. Speech recognition is implemented by exploiting the free, open-source library Vosk, which is an offline recognition toolkit available for 18 languages and dialects. Vosk makes use of artificial intelligence to train a speech recognizer based on language model packages that can be downloaded from the library’s official website. After importing the DLL into the plugins folder, .NET is automatically able to perform the speech recognition by calling the C# delegates of the library. Only English and Spanish models are used in the simulation. The controller activates the microphone if permission is granted, and a compatible device is initiated and starts listening once the user gets near the character that offers a mobile phone, as shown in Figure 6. If the speech recognizer receives a phrase it can understand, it checks if it corresponds to any of the ones saved in the system database. In the case of a correct voice command, a custom event triggers for other components to listen and act in concordance. When calling the emergency services, the call is recorded and saved locally on the computer so it can be evaluated by an instructor later on. A Boolean metric describing whether the user called emergency services is saved for posterior performance evaluation.

#### 2.1.7. Performance Metrics

Performance metrics are generated during the performance of the exercise inside the simulation. The user is assisted by an external mannequin that provides a point of reference in the simulation, as well as tactile feedback. A tracking device is placed on the chin area of the mannequin and is used to locate the victim in the virtual world space regardless of the real workspace of the user who performs the simulation. The 3D model made for the victim allows that the depth of the compressions corresponds to a similar space in the mannequin used, so the simulation is capable of collecting data about (1) the number, frequency, and depth of the repetitions, (2) where the electrodes are located, (3) the number of attempts trying to place them and (4) the time spent in the exercise. Collected data do not require any mechanical devices inside of the mannequin to be measured.

**Figure 7:**
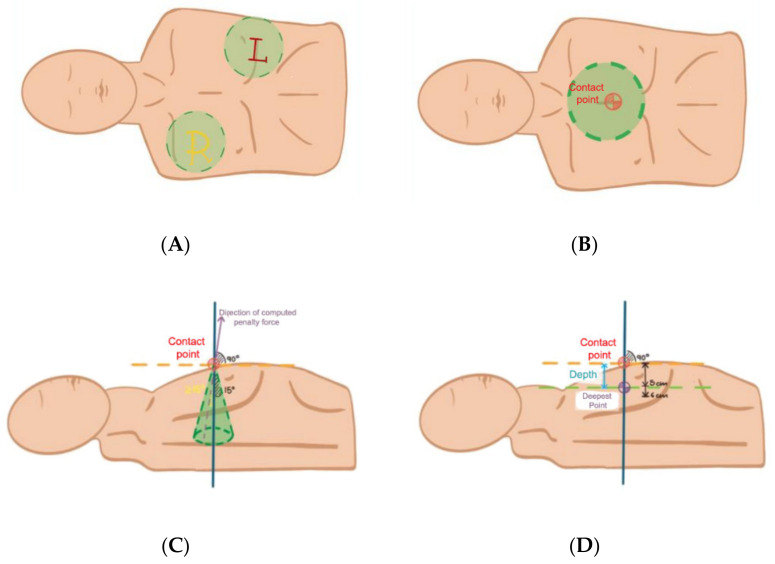
(**A**) Yellow R represents the right electrode location, Red L represents the left electrode location. (**B**) indicates the correct contact point. (**C**,**D**) indicate the direction and deepest point of RCP maneuver.

The electrodes of the AED are virtual objects that the user needs to place inside the correct areas, as shown in Figure 7. The different colors help to identify which electrode can be placed in specific spots. After dropping the electrode inside a threshold bounding the correct area, it snaps in place and cannot be moved again for easier performance. If the user does not place the correct electrode, it respawns inside the AED box. The point of contact to start the compression performance is the area shown in Figure 7B. Within this area, the trainee has to perform CPR with hand tracking or the HTC Vive controller tracking system. As the physical mannequin does not collect any performance data, the metrics are collected based on the force direction vector of the hands and the deformation of the chest mesh in the victim’s body (Figure 7B,C). To evaluate this, the angle of the world-space down vector (vector a) with respect to the applied force of the vector b (direction of the hand movement obtained by the VR tracking system) against the surface of the victim’s 3D mesh, resulting in vector c (normal of the applied force to the closest polygon) and vector c (penalty force to avoid one rigid body to enter the boundings of the 3D mesh); is obtained:

Angle (a, b) = arccos[(xa * xb + ya * yb + za * zb)/(√(xa2 + ya2 + za2) * √(xb2 + yb2 + zb2))](1)

If Angle (a, b) ≤ maximum angle, the inclination is considered correct and counts as a correct compression. However, as no external device blocks the real user movement, there is no movement limitation in the real workspace. Therefore, the location of the hands regarding the action is checked along with every inclination and stored for posterior metric collection. The physical mannequin, on the other hand, offers realistic resistance to the physical movement of the user simulating a real human chest, so the 5–6 cm limit of the depth of the compression is considered in the guidelines of the ERC and is respected [10].

CPR-OSCE VR evaluates the performance in each compression using the position difference of the tracked hands or controllers in different time steps, taking into account a small error threshold where the system can lose track if the HTC Vive stations or the Oculus Tracking Cameras lose sight of the user’s hands as shown in Figure 7D and finally, the performance hit rate is evaluated by:

Hit rate = (Correct compressions/total compressions) * 100 ± threshold (%)(2)

Where threshold is defined by the program as the 5–15% of the complete correct result depending on the configuration file of the trainee before the simulation starts and which is defined by the instructor.

The head is also tracked, so the pause time and the rescue breaths can be evaluated by using the position and rotation of the head in the different time steps and counting the time between compressions greater than 3.5 s as pause time [10]. If the user waits between compressions more than ten seconds, the pause time is considered incorrect. Pause times are stored in temporary files until the end of the simulation, as well as the information regarding whether each compression was correct or not. From these data, metrics about average pause time and hit rate are also calculated. After the simulation ends, the metrics are stored in a .CSV file in the local storage of the running device, under %AppData% on Windows, MacOS, or Linux and Android/OBB on Oculus Quest 2. These metrics need to be analyzed and evaluated by the examiner, who also verifies the correct evaluation of the metrics.

#### 2.1.8. Video Streaming

In some cases, the examiner might desire to follow the simulation performance. Whether CPR-OSCE VR is running on a computer with the HMD connected to it by cable, the trainee sees the 3D environment through the headset, and a second display is shown on the computer’s main screen if it is available. In the case of standalone mode of the Oculus Quest 2, this is not possible due to the VR device being completely wireless and independent. However, the trainee’s performance can be tracked in real-time if the video streaming feature is active while the simulation is running through the settings configuration. Oculus Quest 2 device allows streaming natively the rendered video to casting devices such as Google Chromecast. Nonetheless, this feature requires not only a Wi-Fi connection where the VR and the casting devices are connected but the Google Chromecast device itself. For this reason, a streaming video system was implemented by UDP protocol. When the streaming feature is active, the “center eye” of the VR camera is also rendered to a texture, which is converted to a byte array and sent by UDP to a server if the server is reachable. The end display device must run external software that creates the mentioned server matching the sender’s label, receives those UPD packages, decodes them, and converts the received byte array to a render texture that is displayed on the screen. This feature is completely optional as the examiner can watch the entire performance outside the simulation to evaluate the trainee’s posture while controlling the workspace area to avoid obstacles that might appear while performing.

### 2.2. Validation

The validity of CPR-OSCE VR was studied in a performance battle test. For carrying this test out, the OSCE station was prepared for the HTC Vive with Steam VR plugin support and the Oculus Quest 2, with Oculus XR plugin support, and a mannequin for CPR practice is integrated to give tactile feedback.

After the performance of the test, a Likert questionnaire for collecting qualitative data about the simulation they had to perform was administered to the participants. The questionnaire comprised two yes/no questions, twenty Likert questions about the different parts of the simulation, and seven questions for feedback collection about how to improve the simulator (accuracy, motion sickness, immersion, quality of the CPR maneuver…). The questionnaire was prepared based on different question sets and taking into account the expected profiles. Specifically, questions were divided into four main categories: (1) personal data, (2) previous experience with VR, similar technologies, and OSCE, (3) user experience with CPR-OSCE VR, and (4) fidelity of CPR-OSCE VR regarding OSCE protocol. The actual questionnaire can be found in Supplementary Material S1.

## 3. Results

**Figure 8:**
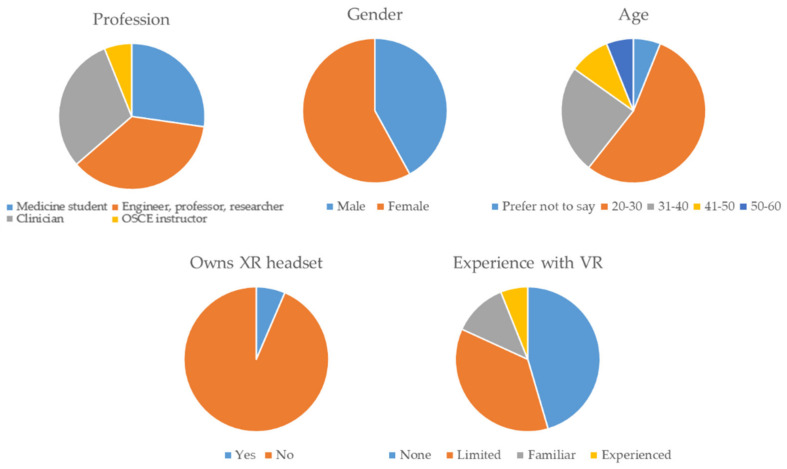
Summary of personal information and familiarity with VR and XR from participants in the validation protocol. Regarding experience: None refers to people that never used VR technology, Limited to people using technology less than once a month, Familiar to people using technology about once a month and experience to people using technology several times a month.

The validation protocol involved a total of thirty-three participants between fifteen healthcare professionals with different medical backgrounds, ten medicine students, six college professors, and two traditional OSCE examiners; aged nineteen to fifty-seven of different nationalities (as summarized in Figure 8) in Hospital La Paz of Madrid. All of them played the same scenario while their metrics were generated. It is worth mentioning that fifteen out of the thirty-three participants had never tested a VR application before, and twelve of them had only played a few VR games in congresses or expositions. However, none of them owned a VR headset. Finally, seven of the participants were used to XR simulations.

Only three percent of the participants found the scenario unnatural or unrealistic enough, and nine percent stated it could be improved. Remarkably, ninety-two percent of the participants that were used to XR experiences found that CPR-OSCE VR met expectations compared to officially launched VR applications in the market, matching the positive feedback of the non-experienced group, based on realism and immersion inside the presented scenario, as well as the interaction with the objects of the scene.

Only nine participants had issues with the VR controllers in the first 2 min. All of them found the simulation a bit long and experienced mental overload during the performance of the maneuver, which may be due to none of them having tested any kind of VR headset or controller before (thus were not used to them).

On the other hand, the tracking system lost track in some frames during the CPR maneuver, losing part of the accuracy while grabbing virtual objects. Despite this, twenty-nine out of the thirty-three participants, including the OSCE instructors, stated that the simulation conforms to ERC protocols and that CPR-OSCE VR can be used for training and evaluating the CPR maneuver performance.

Regarding this, the tracking loss resulted in only forty percent of the participants performing adequate compressions. However, eighty-eight percent of the participants obtained a correct compression rate, and sixty-five percent attained a positive outcome in the appropriated compression depth applied to the mannequin.

**Figure 9:**
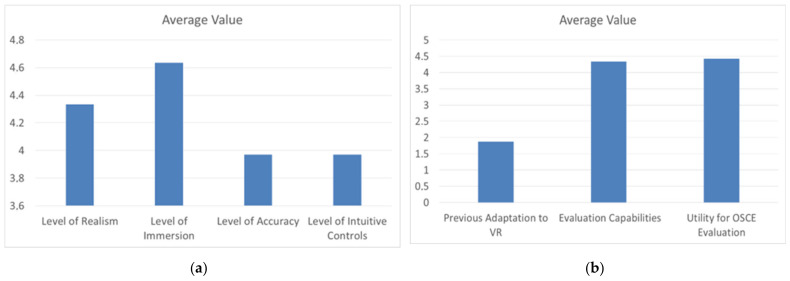
(**a**) Average values of realism, immersion, accuracy and intuition. (**b**) Average of previous adaptation to VR technologies, CPR-OSCE VR evaluation capabilities and utility in OSCE evaluation.

**Figure 10:**
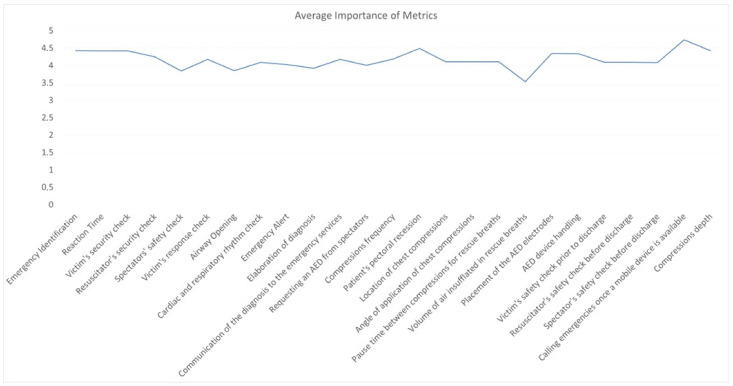
Average values of the importance for each metric mentioned in Table 1.

This proof-of-concept test demonstrated how intuitive and easy to use the simulator is (Figure 9a, and highlighted the importance of engagement in the utilization of VR as a new simulation technique (Figure 9b). Specifically, some participants said that the evaluated metrics are important to the proper OSCE evaluation (Figure 10), and in addition to the fact that if the user is not bored and is engaged in the exercise with new interactive simulations, the performances may have better outcomes.

## 4. Discussion

This software application provides an immersive scenario with an emergency where the student can demonstrate the acquired knowledge and skills regarding the execution of the BLS. The application monitors the performance and provides an objective evaluation at the end of the simulation providing more metrics than the ones commonly offered by standardized feedback devices. The simulation covers the main stages of BLS (i.e., identification and notification to emergency services, use of defibrillator, and CPR maneuver performance).

The resulting system is a tool with the advantages of cross-platform applications that can be mounted in any room, with almost any VR device, and without any need for an Internet connection. As CPR-OSCE VR works natively on PC, it can be used with any VR headset that supports the OpenXR or Oculus XR plugins. Most of them are connected to the computer by cable. However, in the case of using an Oculus Quest to perform the procedure, the simulator works completely wireless, offering a portable experience. The only required action by the instructor is writing the name and ID of the student in the initial configuration file, as well as the duration of the activity. The entire evaluation is carried out automatically by the system, and it follows the same procedure for each student. Therefore, CPR-OSCE VR offers a method of objective evaluation with minimal intervention from the instructor.

The main scene’s design was based on regular off-hospital OSCE station scenarios, so OSCE examiners and examinees could encounter a similar experience while performing in the virtual scenario. The specification of the OSCE station to be off-hospital is especially relevant since there are fewer resources than in the hospital (e.g., manual breathing ballons). The popularization of mobile and VR experiences helps the simulation be more user-friendly as the young participants are usually used to controlling virtual environments with controllers. Regarding the non-accustomed groups, spending an average time of 5–10 min can produce motion sickness and mental overload, so a worse user experience was perceived, not due to the simulation accuracy but the VR itself.

The level of realism and immersion in this application is positive since 97% of participants in the validation trial find the simulation realistic and easy to use. The learning phase at the beginning is the key to this since it is where the students learn and practice the controls and gestures that the students need to use on the main simulation without revealing information about the emergency they are facing. Carrying out these tasks may reduce the students’ learning curve regarding the use of the simulator and the VR controls. In this way, the student’s evaluation is not conditioned by their level of experience with this type of technology.

Additionally, 97% of participants found immersive VR interesting to be applied in other OSCE stations, but only 15.2% of them had tested other OSCE VR stations. This suggests that the approach proposed in CPR-OSCE VR is appealing to new users and could be further exploited commercially.

As expected, the participants who own an XR headset and those who are more used to VR determined that the simulation offers an intuitive experience with habitual triggering controls and low mental overload. On the other hand, those who had never tested any VR devices experienced a tiring sensation and high mental overload after 10 min of the procedure. Furthermore, these participants found frustrating the tracking loss when the two hands overlap during the CPR compressions. This can be solved by using additional tracking cameras that provide more accuracy in the tracking system and performing with a mannequin with the tracking system. Despite this, the main reception of CPR-OSCE VR was mainly positive among the participants. CPR-OSCE VR was found to be the only simulator with the evaluation of the CPR performance in OSCE scenarios which system allows multiple scenarios while collecting data during the whole procedure.

These results show that the developed software achieves all the requirements to be used not only as part of training but also as an assessment tool that analyzes the performance of the student objectively and efficiently, being capable of evaluating OSCE professionals. This is essential since, as stated in the review of commercial solutions, there is a lack of simulators offering evaluation modes with summative and formative feedback. In addition, CPR-OSCE VR is the only one with custom bundle management capable of creating multiple scalable scenarios while maintaining a stable framerate in the standalone mode of the Oculus Quest 2. Most of the presented solutions in the state-of-art make use of the Oculus Rift and regular HTC Vive at 60–72 Hz, while CPR-OSCE-VR makes use of hardware that doubles the refresh rate, allowing smoother rendering and better interpolation between frames while being completely wireless and not requiring a powerful external computer to work. Furthermore, none of the simulators implemented any frame-interpolation techniques such as FSR or AWS. VR mobile applications such as CPR Mobile App VR require to keep a hand holding the cardboard with the device and do not use tracking systems to obtain hand or external controller input, leading to immersion loss. In addition, none of the systems described in the state-of-art have reactive artificial intelligence for simulation events. On the other hand, CPR-OSCE VR’s behavior system offers a reaction to scalable events such as other non-playable characters (NPC) movement, interaction, and event triggering to increase the immersion. Moreover, CPR-OSCE VR can display the performance of the user to external devices even if the simulation is not running through a computer, but the Oculus Quest 2 standalone device only requires a Wi-Fi connection and lightweight software that runs as a server or a Google Chromecast device where the rendered view is streamed to.

However, this simulator is not without limitations. For instance, the Oculus Quest hardware leads to huge limitations in terms of graphic computing and the number of polygons that can be rendered in real-time. If the streaming feature is active while running the simulation in standalone mode, depending on the Wi-Fi connection, battery life percentage, and the number of rendered objects, the framerate is affected. The advantages of cables not being required in standalone mode get overshadowed by the limitation in calculated lightmaps, elements in the scenario, and quality of the textures. This can easily be overpassed by running the simulation on a computer with the headset connected by cable or by a 5 GHz Wi-Fi connection. Furthermore, the hand tracking mode inherently loses track after a few seconds without visualizing the tracked object. This can lead to unexpected errors while performing the CPR maneuver, as the hands might stop being tracked by the headset while they are not in the field of view. The authors are aware that, despite it not being pointed out as a fault by participants in the validation, it should be corrected to improve realism and usability. To tackle this problem, we aim to implement data prediction that can fill the missing information from the hand tracking by interpolating the received hand pose data between frames. The implementation of a virtual coupling scheme between the simulated hand and the tracked hand has given positive results for absorbing large deviations [41]. Another approach to solve this issue is creating a haptic hand tracking system based on a glove capable of tracking rotation and position changes of the hand, as well as each of the phalanx of the fingers. This device would be combined with the implementation of hand-object spatial representation that can achieve generalization from limited data combining the global object shape as voxel occupancies with local geometric details as samples of closest distances [42]. Additionally, the currently used mannequin offers resistance to compressions and gives haptic feedback to the user. However, this mannequin does not support compression depth, rate evaluation or airway opening, and the insufflation is verified by the correct positioning of the participant and the number of decibels caught by the microphone when the participant blows. This can be resolved with hardware inside the mannequin capable of detecting air insufflations, compression depth, rate, and hit rate, sending the evaluated data to the simulation to save it with the rest of the metrics. Initially, the mannequin was intended not only to retrieve realistic feedback (as the user feels the real hardware with its own hands) but to catch and evaluate the mentioned metrics. However, due to issues related to the hardware development, a regular CPR mannequin was used for the simulation.

It is worth mentioning that this validation was designed to be mainly technical (i.e., analyzing technical issues, realism, usability, etc.). However, we aim to further research pedagogical aspects associated with the proposed simulator. Specifically, we propose to analyze skill transfer by repeating the experiment with the same participants six months after the first trial to verify if the practiced maneuver training endures over time. A future multi-user feature is planned so the examiners can connect their own devices over Wi-Fi to participate in the simulation as bystanders and follow the simulation in a more immersive way.

## 5. Conclusions

The development process for CPR-OSCE VR was presented, including its main characteristics, namely (1) immersion in a realistic environment similar to a public place where a cardiac arrest is likely to happen, (2) head and hand tracking and interaction based on physical collisions with hands, and (3) objective data collection and evaluation based on the CPR procedure.

In order to verify the validity of this system, a test was performed with 33 clinicians and OSCE examiners. After the performance of the test, a questionnaire regarding the simulated experience was sent to the participants, and the outcome was analyzed. The obtained outcomes demonstrate that the developed application coordinates the VR elements that the student controls with a virtual environment that represents a real situation in an intuitive and easy-to-use way.

In conclusion, CPR-OSCE VR is one of the best existing solutions for VR OSCE performance evaluation, being capable of collecting objective data during the simulation of the trainee progression while providing a realistic environment combined with an accurate and intuitive procedure. This can be used as an alternative method for evaluating the CPR protocol in OSCE stations that can be repeated as many times as needed without external extra resources or materials. Thus, not only doctors or healthcare training centers, where the resources are greater thanks to additional material such as the manual balloon resuscitators, but out-of-hospital settings could benefit from CPR-OSCE VR but also associations and organizations which include CPR training courses within their offers.

## Figures and Tables

**Table 1:** Metrics obtained in the different systems and the evaluated skill based on the metric obtainment.

Metrics	Units	Evaluated Skill
Emergency identification	Yes/No	Clinical judgement, diagnosis management
Reaction time	Time in seconds	Clinical judgement, diagnosis management
Checking patient, bystander and rescuer safety	Yes/No	Ethical, legal aspects and professionalism
Checking the patient’s response	Yes/No	Physical exploration
Opening of the breathing airway	Yes/No	Technical and procedural skills
Checking heart and breathing rates	Yes/No	Physical exploration
Diagnostic communication to emergency services	Yes/No	Communication skills
Calling to emergency services	Yes/No	Communication skills
Diagnosis elaboration	Right/Wrong	Anamnesis, triage
Request for an automated external defibrillator (AED)	Yes/No	Communication skills
Time to carry out the preliminary checks	Time in seconds	Technical and procedural skills
Frequency of CPR compressions	Number	Technical and procedural skills
Depth of CPR compressions	cm	Technical and procedural skills
Pectoral recoil of the patient	Complete/Incomplete	Technical and procedural skills
Location of CPR compressions	Right/Wrong	Technical and procedural skills
Angle of application of CPR compressions	Right/Wrong	Technical and procedural skills
Pause time between compressions for rescue breaths	Time in seconds	Technical and procedural skills
Volume of air insufflated in rescue breaths	Liters	Technical and procedural skills
Placement of the AED electrodes	Right/Wrong	Technical and procedural skills
Handling the AED device	Right/Wrong	Technical and procedural skills
Checking the patient’s, bystanders’ and rescuer’s safety prior to electroshock	Yes/No	Ethical, legal aspects and professionalism

**Table 2:** Simulators and their collected information.

Simulators	RELIVE	LISSA	CODE-BLUE	VR-UCAM	LIFE SAVER	VR-Hybrid	VR-CPR	CPR Mobile	CPR-VR	FPS CPR VR
Type of simulation	Mini-VREM	Mini-VREM	VR	VR	VR	VR	VR	VR	VR	VR
Immersive	No	No	Yes	Yes	Yes	Yes	Yes	Yes	Yes	Yes
Emergency scenario	No	Yes	Yes	No	Yes	Yes	Yes	Yes	Yes	Yes
Multiple scenarios	No	Yes	No	No	Yes	No	No	No	No	No
Mannequin usage	Yes	Yes	Yes	Yes	Yes	Yes	Yes	No	Yes	No
Learning Mode	No	Yes	Yes	No	Yes	Yes	No	No	Yes	Yes
Training Mode	Yes	Yes	Yes	Yes	Yes	Yes	Yes	Yes	Yes	Yes
Evaluation Mode	No	No	No	No	No	Yes	No	Yes	No	No
Simultaneous users	1	1	1	1	1	1	1	1	1	1
Feedback	I	I	I	I	I	I + E	I	E	I	I + E
Haptic Device	M	M	C	M	M	M	M	C	M + C	C

Immediate (I), end of the simulation (E), mannequin (M) and controller (C).

## Data Availability

Data available on request due to restrictions of privacy.

## Supplementary Materials

The following supporting information can be downloaded at: https://www.mdpi.com/article/10.3390/s22134913/s1. S1: Questionnaire for validation protocol.
