# Supplementary material for: A New Immersive Virtual Reality Station for Cardiopulmonary Resuscitation Objective Structured Clinical Exam Evaluation
Source: Sensors (Basel). 2022 Jun 29;22(13):4913. doi: 10.3390/s22134913 (PMC9269536; doi:10.3390/s22134913)
Supplement: Supplementary file 1 [file sensors-22-04913-s001.zip › sensors-1764598-Supplementary.pdf]

Supplementary material

S1. Questionnaire for validation protocol.

User agreement

1. Do you agree in using the provided information for the purposes of the CPR-OSCE VR validation protocol? **[Yes]**

Personal Data

2. Indicate your gender
   - a. Male
   - b. Female
   - c. Non-binary
3. Age group
   - a. <20
   - b. 20-30
   - c. 31-40
   - d. 41-50
   - e. 51-60
   - f. >60
4. Country **[Open answer]**
5. Professional experience
   - a. Medical student
   - b. Professor
   - c. OSCE examiner
   - d. Healthcare worker
   - e. Other **[Open answer]**

Previous experience with VR, similar technologies and OSCE

6. Please grade, from 1 to 5, your previous experience with VR

User experience with CPR-OSCE VR

7. Please grade, from 1 to 5, your perception on the following aspects:
   - a. Graphics realism.
   - b. Intuitiveness of controls
   - c. Accuracy in the compressions, object grabbing, interacting with the menus and environment.
8. Did you find any bugs or errors during the simulation? **[Open answer]**
9. Please rate, from 1 to 5, the agreement level on these sentences:
   - a. CPR-OSCE VR is immersive and takes advantage of VR.
   - b. CPR-OSCE VR produces motion sickness.
   - c. CPR-OSCE VR produces mental overload.
   - d. CPR-OSCE VR produces physical overload.
   - e. CPR-OSCE VR simulator is useful for CPR skills' training.
   - f. CPR-OSCE VR simulator is useful for CPR skills' assessment.
   - d. CPR-OSCE VR can be considered a useful alternative against traditional OSCE stations.
   - e. Timing of the exercise was appropriate.
   - f. The use of immersive VR in this simulator is well applied.

Fidelity of CPR-OSCE VR regarding OSCE protocol

10. Procedure realism regarding the OSCE protocol. X
11. Grade, from 1 to 5 the following metrics:
   - a. Emergency identification
   - b. Reaction time
   - c. Security checks for the victim, bystanders and user
   - d. Airway opening
   - e. Basic check of the victim vitals
   - f. Emergency alert check
   - g. Correct diagnosis

   h. Communication with emergency services with the results of the maneuver
   i. Related to compressions: number of compressions, compression time, angle applied in each compression, compression depths, compression frequency, location of each compression, pause between compressions,
   j. Volume of insufflated air
   k. Related to AED usage: AED request to the bystanders, AED electrode location, usage of the AED

12. Would you add any other metric? **[Open answer]**
13. Would you consider useful to apply VR in other OSCE stations? **[Yes/No]**
14. Do you know other OSCE station simulators? **[Yes/No]** If so, which one? **[Open answer]**
